# Measuring patient activation in Italy: Translation, adaptation and validation of the Italian version of the patient activation measure 13 (PAM13-I)

**DOI:** 10.1186/s12911-015-0232-9

**Published:** 2015-12-23

**Authors:** Guendalina Graffigna, Serena Barello, Andrea Bonanomi, Edoardo Lozza, Judith Hibbard

**Affiliations:** Department of Psychology, Università Cattolica del Sacro Cuore, Milan, Italy; Department of Statistical Sciences, Università Cattolica del Sacro Cuore, Milan, Italy; Department of Planning, Public Policy, and Management, University of Oregon, Eugene, USA; Faculty of Psychology, Università Cattolica del Sacro Cuore, L.go Gemelli 1, Milan, 20123 Italy

## Abstract

**Background:**

The Patient Activation Measure (PAM13) is an instrument that assesses patient knowledge, skills, and confidence for disease self-management. This cross-sectional study was aimed to validate a culturally-adapted Italian Patient Activation Measure (PAM13-I) for patients with chronic conditions.

**Methods:**

519 chronic patients were involved in the Italian validation study and responded to PAM13-I. The PAM 13 was translated into Italian by a standardized forward-backward translation. Data quality was assessed by mean, median, item response, missing values, floor and ceiling effects, internal consistency (Cronbach's alpha and average inter-item correlation), item-rest correlations. Rasch Model and differential item functioning assessed scale properties.

**Results:**

Mean PAM13-I score was 66.2. Rasch analysis showed that the PAM13-I is a good measure of patient activation. The level of internal consistency was good (α = 0.88). For all items, the distribution of answers was left-skewed, with a small floor effect (range 1.7–4.5 %) and a moderate ceiling effect (range 27.6–55.0 %). The Italian version formed a unidimensional, probabilistic Guttman-like scale explaining 41 % of the variance.

**Conclusion:**

The PAM13-I has been demonstrated to be a valid and reliable measure of patient activation and the present study suggests its applicability to the Italian-speaking chronic patient population. The measure has good psychometric properties and appears to be consistent with the developmental nature of the patient activation phenomenon, although it presents a different ranking order of the items comparing to the American version.

PAM13-I can be a useful assessment tool to evaluate interventions aimed at improving patient engagement in healthcare and to train doctors in attuning their communication to the level of patients’ activation. Future research could be conducted to further confirm the validity of the PAM13-I.

## Background

Western healthcare systems are still geared toward acute care [1] and are not well equipped to handle the long-term commitment required to treat chronic conditions. Patients themselves and their families are a crucial resource to the long-term management of chronic conditions, as they can actively contribute to shape the care process in order to make it more and more aligned to their expectations [2–7]. Optimal treatment of chronic illnesses requires not only a healthcare system that recognizes the patient as central to his or her care; it also requires an activated patient who knows what to do (in terms of health management behaviors and who has the skills and motivation to do so (in terms of attitudes and knowledge). Many researchers [8–10] have found that engaged, informed, confident, and skilled patients are more likely to perform activities that will ensure their own health. Moreover, active patient engagement in healthcare has been also demonstrated to lower healthcare costs [11].

To successfully engage patients in their healthcare, it is fundamental to deeply understand their subjective illness experience and attitudes when navigating healthcare. An experience that is complex at the psychosocial level and that develops in time, according to the features of the patient’s illness journey [12–15]. Tools able to assess and targeting the level of activation and engagement of patients in their health management are crucial, although they are still underdeveloped and scarcely used [16]. According to Hibbard and colleagues [17], patient activation is defined as having “*the knowledge, skills, confidence and behaviors needed for managing one's own health and health care*”. Patient activation describes the level of patients’ engagement in their healthcare and, according to many outcome studies in this field, it leads to better disease self-management [18, 19], higher adherence to medical treatment and greater patient satisfaction [17, 20]. Patients on a higher activation level more often engage in healthy behaviors, more actively cope with their illness, make more efficient use of healthcare services and perform better self-care [21]. Cross-sectional studies have demonstrated that chronically ill and primary care patients who are more actively involved in their care not only have better self-reported health outcomes [19], but also better clinical outcomes [22, 23]. The American Patient Activation Measure short form (PAM 13) [17] consists of 13 items measuring patients’ self-reported knowledge, motivation, and skills for self-management (see Appendix 1). It was developed using a Rasch model [18] and it has been validated in the US general population and, more recently, in other countries – such as Germany [24], Netherlands [25], Denmark [26] Israel [27], Norway [28], and Korea [29] – across different clinical settings [22, 23, 30–32].

Italian policy makers and public health researchers are more and more interested in strategies to effectively make chronic patients actively engaged in their healthcare [13, 14, 33–35]. In Italy, according to the European statistics, this clinical population is growing in numbers and is more and more gaining attention due to its potential social and economic impact [21]. For these reasons, there is definitely a clinical and scientific need for a validated assessment tool that can help practitioners differentiate patients into subgroups that require different strategies in health support, information and communication. Therefore, the present research project was conducted in Italy in order to:

- translate the American short form Patient Activation Measure (PAM 13) into an Italian version (PAM13-I);
- establish the psychometric properties of the Italian version of the PAM 13; and
- validate the Italian version of the American PAM 13 in a sample of chronically ill patients.

## Methods

### Recruitment and data collection

This cross-sectional study included a simple random sample of 529 Italian-speaking adults affected by different chronic conditions. Patients were randomly selected and recruited through the online panel provided by Research Now (http://www.researchnow.com/en-US.aspx), a professional research institute with branches across the world. The panel covers a wide range of chronic conditions and counts more than 6.5 million registered subjects worldwide. Subjects belonging to the panel are carefully screened for authenticity and legitimacy via digital fingerprint and geo-IP-validation from the provider. Panel recruitment and data collection processes are compliant with national laws in each country where Research Now operates. All panelists are profiled on the basis of their socio-demographic, clinical and life-styles characteristics. The panel is certified to be statistically representative of all the covered populations. In our study, in order to guarantee data quality, respondents were asked to confirm their demographics (i.e. sex, date and place of birth, ethnicity, nationality, educational level, place of residency) and clinical condition (i.e. health status, chronic diagnosis, date of first diagnosis, prescribed medications) previously collected by the Research Now Panel. To be included in our study, patients had to be Italian, affected by one or more chronic conditions, aged over 18 years old, and of both genders. Patients with dementia, cognitive impairments, active psychiatric disorders, blindness, deafness, or insufficient Italian language skills to meaningfully answer to the questions or without informed consent were excluded from this study. All participants gave written informed consent before being enrolled in the study. Patients completed the PAM13-I questionnaire between October and December 2014. Ethic approval was obtained from the Ethics Committee of the Università Cattolica del Sacro Cuore, Milan (Italy).

### Translation and cultural adaptation of the American PAM 13

After receiving permission from Insignia Health, Inc., PAM 13 - the American original version - [17] was translated as recommended by the World Health Organization’s procedures for cross-cultural validation and adaptation of self-report measures [36]. This method includes the following steps: forward translation, experts’ qualitative interviews, backward translation, pilot testing on patients (for checking the readability and understanding of items) and consensus about the final version (see Appendix 2). The forward and backward translation were performed by professionals who were familiar with the lexicon of the field, knowledgeable in both English and Italian cultures. A bilingual expert panel, composed by twelve individuals (experts in chronic care, health researchers, clinicians and translators), was convened to identify and resolve ambiguous expressions or concepts that could lead to misunderstanding. Discrepancies were discussed, consensus was achieved, and the cultural appropriateness of the translation was confirmed. Finally, a pilot testing of the scale was performed on fifteen chronic patients to investigate their understanding of the items and cognitive equivalence of the translation, followed by debriefing. PAM13-I was judged clear and acceptable and the final version was created by consensus.

### Measures

#### PAM13-I

PAM13-I consists of 13 items on a Likert scale. According to the American version of PAM 13, each item has five response categories with scores from 1 to 5: (1) “Strongly Disagree”, (2) “Disagree”, (3) “Agree”, (4) “Strongly Agree” and (5) “Not Applicable”. The instrument design reflects the four stages of activation in a progressing difficulty of the items: level 1 (patients believe that their role is important: item 1 and 2), level 2 (patients have confidence and knowledge to take action: items 3–8), level 3 (taking action: items 9–11) and level 4 (staying on course under stress: items 12 and 13). According to Insigna Health Inc. guidelines, the raw scores were transformed through natural logarithm to achieve a better expression of the relative distance between the scores. Then, items were transformed to a standardized metric ranging from 0 to 100 (0 = lower activation; 100 = highest activation), to compare Italian results to the original data. The score was calculated by summing up the raw scores and mapping up the sum onto a scale of 0–100. A higher score of PAM13-I indicates a high level of patient activation.

#### Other measures

Age, gender, chronical disease (Asthma, Celiachia, Hypertension, Chronic Obstructive Pulmonary Disorder, Diabetes, Cardiovascular Disorder, Cancer, Chron, Fibromialgy, Coliteulcerosa, Lupus, Osteoatritis, Artritereumatoide, Hypercolesterolemia, Epatitis, Anemy, Allergy), marital status (divorced, married, single, widow, and widower), education (graduated, high school, middle school, and primary school), profession (employee, freelancer, student, retired, unemployed) and children (yes or no) were used as background variables.

### Data analysis

According to COSMIN checklist [37] and to previous PAM 13 validation studies [24, 26], the statistical analysis was conducted in three main steps: missing data analysis, reliability analysis, and Rasch Model analysis.

The first phase regarded the data quality analysis. According to other validation studies [24, 25], participant who filled out less than 7 items on the PAM13-I questionnaire were excluded from validation study. Data were described for each item with frequencies and percentage of missing responses, response options (“Strongly Disagree”, “Disagree”, “Agree”, “Strongly Agree”, “Not Applicable”) and with several statistical indices (mean, median, standard deviation). An evaluation of floor and ceiling effects was also performed.

The second step regarded the reliability analysis. Internal consistency and reliability analysis were assessed using Cronbach’s α as well as item-rest correlation, inter-item correlation, average inter-item correlation. Cronbach’s α of 0.80 was defined as acceptable [38]. Item-rest correlation provided empirical evidence that each item was measuring the same construct measured by the other items included. A correlation value more than 0.3 indicates a moderate and valid correlation with the scale overall and, thus, the item should be not removed [39]. Average inter-item correlation is a subtype of internal consistency reliability, obtained by taking all of the items on a test that test the same construct, determining the correlation coefficient for each pair of items, and finally taking the average of all of these correlation coefficients. This final step yields the average inter-item correlation. An average inter-item correlation between 0.15-0.50 was considered acceptable [38].

In the third step, a Rasch Model (RM) was implemented to examine the PAM13-I psychometric properties. RM is useful to investigate unidimensionality of the construct (fundamental requisite of the summarization of the raw scores), the fit and the reliability of each item, and the differential item functioning.

The Rasch model assumes that the responses are affected by two different components that work independently [40]. The first component concerns the individual characteristics of the subjects and the other to the “displacement” of the generic item gathering the latent aspect of interest. The classical approach of RM [41] assumes that the response probability of each subject to a generic item depends on the level of the latent aspect (ability) and on the difficulty of the item. RM belongs to the family of IRT measurement models, which scale raw observed scores into linear reproducible measurements. Under the hypotheses that there are two different aspects (linked to subjects and items), acting in a separable manner, RM allows for constructing a single metric scale defining a ranking of Items and Person parameters. RM is designed to estimate the subject’s level on the latent trait, net of item characteristics, and items’ net of subjects. Let *p*_*ijk*_ be the probability that unit *i*, with person parameter *θ*_*i*_, chooses the category *k* for evaluating the item *j*; it may be represented through a proper “link function” *φ* (*θ*_*i*_*,β*_*j*_) in the parameters *θ*_*i*_ and *β*_*j*_, accounting, respectively, for personal and item characteristics. RM assumes the last relation to be of the logistic type. In the family of the polytomous models, we consider the Partial Credit Model (PCM) to our sample to examine model-data fit. PCM was chosen because the PAM13-I items had more than two response options and they showed different pattern of usage. Since it is reasonable to assume that the thresholds are not the same for all the items, i.e., each item has its own unique rating scale structure, the PCM appears the most appropriate model. The parameters of the model are estimated by the maximum likelihood method [41]. We performed PCA - part of PCA on Rasch residuals - in order to test the unidimensionality of the construct under investigation.

In the context of patient activation measure analysis, the parameters *θ*_*i*_ and *β*_*j*_ have a specific interpretation. The individual characteristic *θ*_*i*_, usually called “ability”, may be conceived as the individual activation: subjects with higher score in this subscale (Personal Location) will have a higher level of activation. The item characteristic *β*_*j*_, called “item difficulty” in the classical Rasch example, in this context represents the item propensity to obtain, by the respondents, systematically high or low scores when measuring the latent trait of interest. They reflect the level of relevance for a particular aspect measured by each item of the survey. In this way, it is possible to order the survey’s items basing on their different tendency in arousing the activation.

Unidimensionality of items composing PAM13-I was examined, using a Principal Component Analysis (PCA). The aim is to obtain one common factor that explains at least 30–40 % of the total variance. The other fundamental condition is that there are no other factors having eigenvalues greater than 3 [42]. Bartlett’s test of sphericity was performed to explore the factorability of the correlation matrix and Kaiser-Mayer-Olkin Index was calculated as a measure of sampling adequacy.

Local Independence of item was tested by a PCA conducted on the Rasch item measure residuals, in order to analyze the amount of unexplained variance and whether this unexplained variance indicates that there may be more than one dimension. Generally, Rasch-conforming data produces residual-factors with eigenvalues up to 2.0 [43]. Thus, if there is more than one contrast (factors) in the residuals, there may be a second dimension. Contrasts in the Rasch analysis of residuals contradict unidimensionality.

Two item fit mean square (MNSQ) statistics (infit and outfit) were computed to check whether the items fitted the expected model. MNSQ determines how well each item contributes to defining a single underlying construct. Infit is more sensitive to misfitting responses to items closest to the person's ability level, while outfit is more sensitive to misfitting items that are farther away. If the data fit the Rasch model, the fit statistics should be between 0.6 and 1.4. According [44], for clinical observations, the fit statistics could be between 0.5 and 1.5 [26].

The person separation index (PSI) is indicators of quality of measures. The PSI refers to the reproducibility of the measure location of the persons. A separation index, in its normalized form, of 0.80 or higher is considered reliable.

Differential Item functioning (DIF) was assessed in order to verify no relevant differences in the probability to endorse a certain item for subgroups, determined by gender, age and education. The scale should work irrespective of the group considered. Andersen’s Likelihood Ratio Test was performed for each item. To evaluate floor and ceiling effects we evaluated for each item the percentage incidence respectively of the lower level of the scale (*Strongly Disagree*) and of the upper one (*Strongly Agree*). Analyses were conducted with IBM SPSS 21.0 and R 3.0.3 (package eRm).

## Results

### Translation and adaptation

During the translation and forward translation process, we recognized few general problems when comparing the Italian version with the American one [17]. As in the forward-translation, we had difficulty translating health service terminology, partly because of organizational differences, and partly because of a lack of specific Italian words for “healthcare”, “illness” and “disease”. The instrument pre-testing administered to patients in a preliminary phase of the study, allowed understanding that some terms - i.e. “health” and “disease”- might have different meanings and conceptualizations for Italian patients. For example, in item 2, the American form of PAM 13 uses the terms “health” and “ability to function”, referring to the capability of a patient to reach positive outcomes by having an active role in his health management. According to the results of the translation process, researchers decided to use “*benessere*” [= “wellbeing” instead of “health”] and “*qualità di vita*” [= “quality of life” instead of “ability to function”] in the final PAM13-I. This change in translation is a consequence of the fact that, for the Italian patients, “health” means a general state of wellbeing that is not a mere absence of an illness condition. Another case of linguistic adaptation occurs in item 9, where “health” was traduced with “disease” [= *malattia*]. The reason behind this linguistic choice was that, referring to a medical treatment, Italian subject are more akin to think about a clinical condition, and thus referring to “disease”.

### Study Population

Among 600 individuals who were contacted, 529 participants completed the survey (redemption rate: 88 %), which elicited socio-demographic and clinical data, including the PAM13-I scale. Participant who filled out less than 7 items on the PAM-I questionnaire were excluded from validation study. According to inclusion/exclusion criteria, the final sample suitable for the study included 519 patients. The main socio-demographic characteristics of the sample included in the study are described in Table 1.

**Table 1:** Mean PAM13-I scores by socio-demographic and health characteristics

Characteristics	Total N	%	PAM 13 Score Mean	*P*-value
Total	519	100	66.2	
Sex				0.14
Male	255	49.1	67.3	
Female	264	50.9	65.1	
Age Group				0.02
<45	170	32.8	68.1	
45–64	212	40.8	66.8	
>65	137	26.4	62.9	
Education				0.10
Primary school	81	15.6	63.3	
Middle school	88	17.0	64.6	
High school	226	43.5	66.5	
Graduate or higher	124	23.9	68.8	
Diagnosis				-
Asthma	85	16.4	64.3	
Celiachia	13	2.5	74.9	
Hypertension	105	20.2	66.3	
Chronic Obstructive Pulmonary Disorder	21	4.0	61.5	
Diabetes	84	16.2	66.5	
Cardiovascular_Disorder	151	29.1	68.2	
Oncology	109	21.0	65.1	
Chron	9	1.7	64.5	
Fibromialgy	27	5.2	58.3	
Coliteulcerosa	16	3.1	65.8	
Lupus	8	1.5	69.6	
Osteoatritis	38	7.3	60.6	
Artritereumatoide	38	7.3	65.6	
Hypercolesterolemia	53	10.2	62.9	
Epatitis	11	2.1	62.1	
Anemy	15	2.9	71.6	
Allergy	86	16.6	68.9	

Patients aged from 20 to 90 (M = 53.0, SD = 17.1). Gender was equally distributed (49.1 % male, 50.9 % female), with different educational levels. Regarding health characteristics, patients reported having at least one of chronic disease. The most common clinical conditions were asthma (16.4 %), hypertension (20.2 %), diabetes (16.2 %), cardiovascular disorder (29.1 %), cancer (21.0 %), and allergy (16.6 %). The majority of the patients included in our sample was affected by more than one chronic condition.

### Description of the PAM13-I

Table 1 shows the mean PAM13-I scores. Raw scores range from 13–52. These are converted to “activation scores” ranging from 0–100 [45], which can be analyzed as a continuous variable. Higher scores indicate increasing patient activation.

In the Italian sample, male patients had a slightly higher level of activation, but this difference was not significant (*p* = 0.14). Moreover, no significant differences were found in the mean PAM13-I score based on education (*p* = 0.10), although PAM13-I score increased in relation with educational level. Concerning the participant’s age the sample was clustered in three age groups (<45 years old, 45–64 years old, > 65 years old). With increasing age, the level of activation significantly decreased (*p* = 0.02). For this factor (diagnosis), a test was not conducted, since almost all the participants had more than one disease and, consequently, the groups were not independent.

### Validation study

#### Psychometric properties

To evaluate the psychometric properties of the PAM13-I, data quality, internal consistency, reliability, item-rest correlation, item-total correlation and average inter-item correlation were assessed. Table 2 describes the psychometric features of the 13 items included in the PAM13-I.

**Table 2:** Data description of the Italian version of the PAM13 (PAM 13-I)

PAM13-I item no.	Missing Values n (%)	Not Applicable n (%)	Strongly Disagree n (%)	Disagree n (%)	Agree n (%)	Strongly Agree n (%)	Median	Mean	SD	Inter-rest correlation
PAM13-I 1	4 (0.8 %)	2 (0.4 %)	15 (2.8 %)	47 (8.9 %)	170 (32.1 %)	291 (55.0 %)	4	3.41	0.77	0.36
PAM13-I 2	5 (0.9 %)	5 (0.9 %)	9 (1.7 %)	45 (8.5 %)	184 (34.8 %)	281 (53.1 %)	4	3.42	0.72	0.41
PAM13-I 3	4 (0.8 %)	11 (2.1 %)	11 (2.1 %)	48 (9.1 %)	238 (45.0 %)	217 (41.0 %)	3	3.29	0.72	0.53
PAM13-I 4	6 (1.1 %)	12 (2.3 %)	15 (2.8 %)	44 (8.3 %)	209 (39.5 %)	243 (45.9 %)	3	3.33	0.76	0.62
PAM13-I 5	4 (0.8 %)	8 (1.5 %)	15 (2.8 %)	68 (12.9 %)	234 (44.2 %)	200 (37.8 %)	3	3.20	0.77	0.60
PAM13-I 6	5 (0.9 %)	4 (0.8 %)	14 (2.6 %)	44 (8.3 %)	210 (39.7 %)	252 (47.6 %)	3	3.35	0.75	0.60
PAM13-I 7	5 (0.9 %)	7 (1.3 %)	17 (3.2 %)	42 (7.9 %)	196 (37.1 %)	262 (49.5 %)	4	3.36	0.77	0.55
PAM13-I 8	11 (2.1 %)	8 (1.5 %)	15 (2.8 %)	79 (14.9 %)	225 (42.5 %)	191 (36.1 %)	3	3.16	0.79	0.55
PAM13-I 9	6 (1.1 %)	14 (2.6 %)	24 (4.5 %)	78 (14.7 %)	234 (44.2 %)	173 (32.7 %)	3	3.09	0.82	0.54
PAM13-I 10	8 (1.5 %)	9 (1.7 %)	15 (2.8 %)	43 (8.1 %)	244 (46.1 %)	210 (39.7 %)	3	3.27	0.74	0.58
PAM13-I 11	6 (1.1 %)	9 (1.7 %)	16 (3.0 %)	68 (12.9 %)	247 (46.7 %)	183 (34.6 %)	3	3.16	0.77	0.65
PAM13-I 12	6 (1.1 %)	4 (0.8 %)	21 (4.0 %)	107 (20.2 %)	245 (46.3 %)	146 (27.6 %)	3	2.99	0.81	0.66
PAM13-I 13	4 (0.8 %)	11 (2.1 %)	16 (3.0 %)	71 (13.4 %)	256 (48.4 %)	171 (32.3 %)	3	3.13	0.76	0.58

Percentage of missing values per item was low and it ranged between 0.8 % (items 1, 3, 5, 13) and 2.1 % (item 8), as well as percentage of “Not Applicable” (ranging between 0.4 % and 2.6 %).

For all items, the distribution of answers was left-skewed, with a small floor effect (range 1.7–4.5 %) and a moderate ceiling effect (range 27.6–55.0 %).

The frequencies of the response categories showed little use of the category “Strongly Disagree” and a moderate frequency for “Disagree”. The categories “Agree” and “Strongly Agree” were used with the highest frequency for all the items.

The median score was 4 for items 1, 2 and 7, while for all the other items the median score was equal to 3. The mean scores ranged from 2.99 (item 12) to 3.42 (item 2). Standard deviations showed a constant variability in all items, ranged from 0.72 (items 2, 3) to 0.82 (item 9).

Generally, the last items have a lower mean score compared to the earlier items in the questionnaire. However, in our results the individual item sequence did not exactly follow the original US scale (from higher to lower scoring items).

A Cronbach’s alpha equal to 0.88 for the sum scale and a Person Separation Index of 0.89 were found, and they were considered as a very good level of internal consistency. The inter-rest correlations per item to the sum scale were moderate (for item 1 and 2) to strong (for all other items) and ranged from 0.36 to 0.66. The inter-item correlations ranged from 0.16 to 0.56, with an Average inter-item correlation equal to 0.36. All reliability indices supported the assumption of unidimensionality of the measured construct, and they were coherent with the results obtained in other validations of PAM 13 [24, 26, 29].

#### Rasch analysis

Unidimensionality is fundamental for the Rasch Model. A PCA on PAM13-I items was conducted with this aim (Table 3). A total of 41.0 % of the variance was explained by one factor with an eigenvalue of 5.33. The correlation matrix had good factorability, Bartlett’s test of sphericity showed the chi-square was significant at the .0001 level (Chi-square = 2129.7, df = 78, *p* < 0.001) and the index of Kaiser-Mayer-Olkin measure of sampling adequacy was equal to 0.89.

**Table 3:** PAM13-I item fit statistics

PAM13_I Item no.	Location	Threshold 1	Threshold 2	Threshold 3	SE	Infit Value MSQ^a^	Outfit Value MSQ^b^
PAM13-I 2	0.05	−1.10	−0.24	1.49	0.4	1.14	1.19
PAM13-I 1	0.25	−0.50	−0.12	1.37	0.3	1.22	1.32
PAM13-I 6	0.31	−0.52	−0.36	1.81	0.3	0.86	0.78
PAM13-I 3	0.31	−0.87	−0.36	2.16	0.3	1.00	1.15
PAM13-I 7	0.35	−0.24	−0.36	1.66	0.3	0.88	0.88
PAM13-I 4	0.36	−0.44	−0.34	1.86	0.3	0.84	0.90
PAM13-I 10	0.45	−0.41	−0.50	2.24	0.3	0.89	0.89
PAM13-I 5	0.52	−0.80	0.06	2.31	0.3	0.86	0.89
PAM13-I 8	0.53	−1.02	0.26	2.35	0.3	0.92	0.96
PAM13-I 11	0.60	−0.73	0.02	2.50	0.3	0.76	0.74
PAM13-I 13	0.66	−0.75	0.06	2.66	0.3	0.91	0.93
PAM13-I 9	0.81	−0.38	0.25	2.56	0.3	1.02	1.14
PAM13-I 12	0.88	−0.85	0.60	2.90	0.3	0.85	0.85

^a^Infit MSQ reflects the similarity of observed responses from model expected response. Being most sensitive when the item and respondent are close together on the activation scale

^b^Outfit MSQ is reflects unexpected observations with respect to the respondent’s other responses. It is most sensitive when the item location on the scale is far away from the person’s location on the scale

**Fig. 1:**
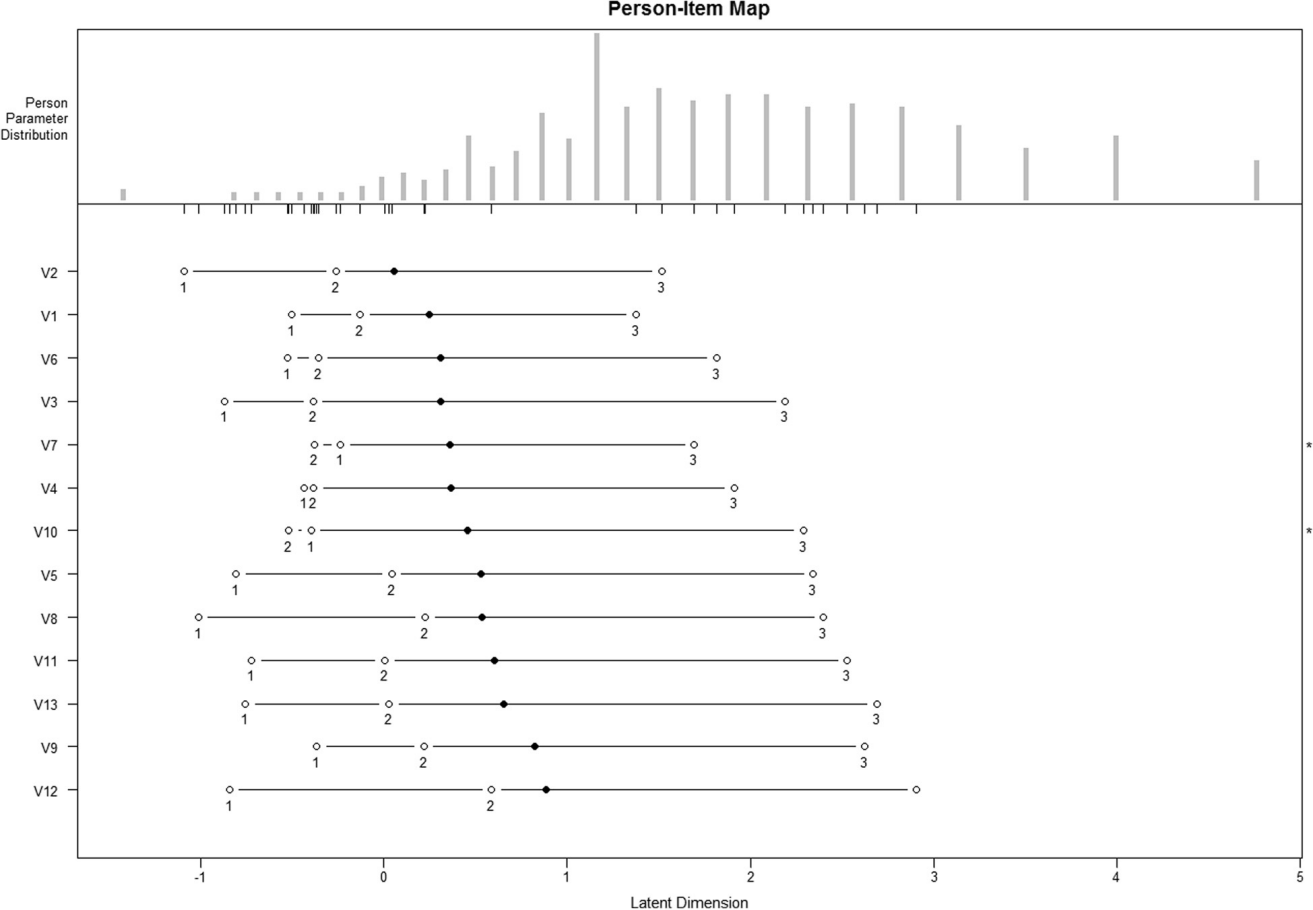
Person-item map

A PCM was used in order to evaluate the item fit statistics. The item statistics ranged from 0.76 to 1.22 for the infit MSQ and from 0.74 to 1.32 for the outfit MSQ. These values indicate a very good fit of the Rasch Model. The distances between subsequent thresholds showed sufficient distinction between the response options and measurement model fit and none thresholds exceeded the limit of 4.0 logit. Only items 7 and 10 presented a minor problem of distinction between thresholds 1 and 2. In Table 3, the item difficulty was ranked according to their location parameters. Results showed that the original ranking of difficulties established for the original American version [17] could not be confirmed for the Italian PAM 13 (the order of the original item corresponds to the difficulty ranking in the American version). The most important differences are related to item 5, 6, 9 and 10. The Rasch person-item map shows the person parameter distribution on the latent underlying dimension and the item difficulties (Fig. 1). Persons on the right side of the scale report being more activated than persons on the left side. Since the black dot represents the location parameter, items with the black dot on the right side of the scale report being more difficult than items with black dot on the left side. White dots represent the thresholds values.

PCA of item measure residuals revealed one dimension. A total of 63.4 % of the variance in the data was explained by the measures and with a perfect model fit, this was expected to be 63.1 %. The eigenvalue of the first PCA contrast was 1.78, which corresponded to 14 % of the variance in the data, the second was 1.65, which corresponded to 12 % of the variance in the data. Rasch dimensionality analysis confirmed the hypothesis of unidimensionality and local independence of the 13 items.

#### Differential Item Functioning

DIF was assessed to reveal significant differences in the interpretation of the items for demographic characteristics (i.e., sex, age, education). The DIF test for sex showed statistical differences between male and female patients in the items interpretation (LR-value = 67.8, df = 38. *p* = 0.002). Differences were more relevant for the interpretation of items 7, 12 and 13, which were slightly more difficult to endorse for female than male subjects were.

The DIF was tested for two subgroups in age (younger than 55 years and older than 55). The test revealed significant differences in items’ interpretation (LR-value = 108.0, df = 38. *p* < 0.001). In particular, item 2,3,6,7 and 13 were more difficult to endorse for the older age group.

Finally, the DIF was tested for education. Two subgroups were compared: the first, in which are collapsed primary and middle school, and the second in which are collapsed high school, graduate and post-graduated. The test showed significant differences (LR-value = 97.6, df = 38. *p* < 0.001). Item 1 and 4 were more difficult to endorse for low education group. Item 7 was more difficult to endorse for high education group. In all cases, DIF was particularly relevant for the third cut off value between the response options “Agree” and “Totally Agree”, but generally it was not so relevant to affect the validity of the instrument.

## Discussion

This study assessed the psychometric properties of the Italian translation of the original American PAM 13. Reliability, validity and responsiveness are context-specific attributes and an instrument that has demonstrated satisfactory measurement properties in one population is not necessarily appropriate for use in other populations [46]. The validation of the PAM 13 into different languages, clinical populations and cultural settings will enable the generalizability of the measure as well as allowing comparisons between countries and the evaluation of context-related health models.

PAM13-I works very well as a measure of activation with all items having very good psychometric properties and item difficulty calibrating very similar to the original PAM 13.

We obtained a high response rate with low percentage of missing values per item. This may minimize the risk of selection bias. The sample size of 519 subjects was sufficient for this validation study as a minimum of 300 respondents is recommended to replicate structural analyses [47]. The mean square statistics used in the Rasch analysis are moderately insensitive of sample size for polytomous data [48].

Findings showed a good internal consistency of the Italian version of the PAM 13 (Chrombach’s alpha: 0.88) which is comparable to the German, Danish, Korean and Dutch version of the instrument and slightly higher than the internal consistency of the Hebrew one (0.77). For the American and Norwegian versions of the instrument, no Chrombach’s alpha was published.

The frequencies of the response categories in our study showed little use of the category “Strongly Disagree” and a moderate frequency for “Disagree”. The categories “Agree” and “Strongly Agree” were used with the highest frequency for all the items. This finding is similar to the one obtained in the German and in the Danish validation studies. The Dutch, Norwegian, Korean and Hebrew validation study did not published this data. The irregular use of response options in Italy, Germany and Denmark could be interpreted as a lack of fit of the response scale in the European patient population.

The mean PAM13-I score (66.2) was higher than the reported score in the original validation study of Hibbard and colleagues (61.9) in the Dutch validation study (61.3), in the Danish (64.2) and in the Norwegian ones (51.93) . On the contrary, the mean PAM13-I score was lower than the German (68.1) and the Hebrew ones (70.7). This might be related to the different samples’ characteristics and to their heterogeneity in term of diagnosis. For instance, the higher rate of activated patients in the Italian sample might be related to their mean age (53 y.o.), lower than the mean age of the German (67 y.o.), Danish (62 y.o.) and Dutch (59 y.o.) patients. Furthermore, the higher rate of activated patients in the Italian sample may be also due to the type of diagnosis: the Italian sample included a wide range of chronic conditions that are recognized to require very different care prescriptions ranging from routine examination to strict medication-taking regimens. Moreover the more easily accessibility of healthcare services in Italy compared to the other healthcare system implies that every citizen is fully covered for basic health services: the gratuity of healthcare services in Italy may make patients perceive them as more easily accessible. The low cost of healthcare in Italy, in other terms, may be considered as an organizational and contextual facilitator of patients’ use of healthcare services and thus of their activation in self-management, but this assumption need further investigation.

Our results also points out that older patients reported lower activation scores. This result confirms previous research evidences that observed the lowest PAM scores among older chronic patients [19, 25, 26]. This finding is not surprising, since previous studies have revealed a lower perceived self-efficacy in older patient compared to a younger clinical population [17, 22]. As people age they are exposed to an increasing variety of personal and social conditions that challenge their sense of control and independence. The tendency for individuals with low perceived self-efficacy to engage in fewer health-promoting behaviors becomes stronger in older adults mainly because physical decline is often viewed as an unalterable part of the aging process [49]. Moreover, older patients have been found to have lower health literacy [50], a factor that can hinder the elderly perception of being successful in self-care tasks. Evidence demonstrated that older patients may be reluctant to seek help for their complaints, they experience more difficulty in seeking and obtaining information during medical interviews and participate less in their medical consultations than other patients, even though they often have multiple health problems [51]. Moreover, age-related factors might hinder the patient’s ability to feel autonomous in his/her daily tasks [52] also for what concerns the medication-taking, thus making them less akin to consider themselves successful in adhere to medical treatments at home. Finally, we can also hypothesize a sort of “generation effect”: older people are more used to a paternalistic healthcare system and they could be less disposed to be active agents in their healthcare.

PCA and Rasch analysis indicated a homogenous factor structure with all items loading on one factor, thus confirming the unidimensionality of the PAM13-I according to the American PAM 13 version. In particular, PCA showed good correlation among items but the explaining variance (41 %) is not so high thus suggesting the possibility that other latent factors could be included but not statistically significant. This finding is similar to the explaining variance calculated in the German study (40.9 %) and in the Norwegian one (37.94 %) thus suggesting that additional factors explaining variance may be considered. This hypothesis is also embraced by studies carried on the neurological population [22, 31, 53]. The latter study performed a confirmatory factory analysis and suggested a tri-factor solutions as the optimal one. The fact that PAM may have a multiple factors structure may open to a conceptual refinement of the concept of activation. Particularly the role of patients’ emotions and of their psycho-social elaboration of the illness’ burden may result worthy to be further explored, with relevant implications for the clinical practice [15].

Furthermore, the Rasch analysis of the items showed that the original difficulty ranking order of the items is not confirmed, similarly to the German, Danish and Dutch validations. The differences in the items order indicate that in the Italian population some items resulted easier or more difficult to respond to than the American population. For example, item 6 was rated as slightly easier than items 3, 4 and 5; and item 10 was rated as slightly easier than items 5, 8 and 9. On the contrary, item 5 was rated as slightly more difficult than items 6, 7 and 10; and item 9 was rated as slightly more difficult than items 10, 11, and 13. Since German, Dutch, Netherlands and Danish validation studies cannot confirm the original items order, this appears to be related to European specificities in the healthcare system and in the chronic populations approach to self-care. Particularly, comparing our findings with the other European validation studies, we can envisage some similarities. For example, item 5 resulted more difficult in comparison to the original order of the American version also in the Danish sample; items 12 and 13 were rated as the most difficult items among Italian, Dutch and German populations consistently with the American one, although in a slightly different order.

Furthermore, differences in the items ranking order between European and American versions appeared as less significant than those highlighted for the Korean validation study, which deeply revised the original American items’ order. This may be due at least partially to the different socio-cultural approach to lifestyles and health management connected respectively to individualistic or collectivistic tendencies [54].

It is also interesting to note some differential items functioning according to gender, age groups and education. According to the results of the DIF test, items 2, 3, 6, 7 and 13 resulted to be more difficult to be endorsed by the subgroup of older patients. This finding is coherent with evidences emerging from the German, Korean and Danish validation studies, which envisaged some differences in the DIF analysis per age related sub-groups. Furthermore, items 7, 12 and 13 resulted to be more difficult to be endorsed by the female subgroup. This finding is partially coherent with those achieved in the German validation study, which registered some differences in the DIF analysis per gender group. Finally items 1 and 4 resulted to be more difficult to be endorsed by the lower educated group while the item 7 resulted to be more difficult ot be endorsed by the higher educated group. This findings are in some extent consistent with those achieved in the Danish validation study, which envisaged some differences in the DIF analysis per education groups. The Dutch, Norwegian and the Hebrew validation study did not performed the DIF analysis. The presence of DIF may suggest a not fully functionality of the scale items in the PAM13-I, particularly for the item 7 (“*I am confident that I can follow through on medical treatments I need to do at home”*) and 13 (“*I am confident that I can maintain lifestyle changes like diet and exercise even during times of stress”*) across the considered subgroups of subjects. However, since all the other psychometric properties of the PAM13-I has been demonstrated to be acceptable we consider that the DIF results do not compromise the validity of the instrument.

Concerning this study’s limitations, the rather heterogeneous group of patients may be regarded as a weakness. However, when assessing scale properties, the use of a population representing many levels of activation could be considered an advantage. Furthermore, although the sample included in our study is not a stratified and fully representative of the Italian chronic population, it was randomly selected in order to guarantee its probabilistic feature. We used it only to explore the relationships of the variables under analysis (i.e. for associative purposes and not for a descriptive estimation of their dimensions): based on these considerations full representativeness is not necessarily required [55, 56]. However, even though population parameters were not the study aim, the quality of the study might be negatively affected and also create errors in the current analysis, thus further research is warranted to confirm the validation of the PAM13-I. Limitations of this study relate to the cross-sectional design that does not allow for calculation of test-retest reliability because we only have one measurement point. Furthermore, similarly to the Korean, Danish and the Norwegian validation studies of PAM 13, our study did not include correlation measures to test construct and criteria validity. However construct and criteria validity were previously tested in the first development and validation process of the America version of PAM 13, by well demonstrating the validity of the instrument [17].

## Conclusion

It can be concluded that the aims of the study were achieved, especially considering that the validity and reliability of the PAM13-I were measured through rigorous and extensive methodological analysis. As it allows the adaptation of instruments to the specificities of healthcare systems and care process that are culturally rooted, the importance of conducting cross-cultural validation studies should be emphasized. Moreover, the psychometric findings presented provide more credibility to its use, both as a methodological resource in research and as a screening tool in a wide variety of clinical context. Assessing patients’ activation level could be crucial in order to train health practitioners in effectively communicating with their patients and to tailor health messages and self-management goals, so relevant when treating chronic diseases [34, 57, 58]. Compared to the regular clinical approach, an intervention with tailored messages has proven to lead to greater improvement in the patients’ clinical markers, such as enhanced adherence to prescribed medication regimens, shorter hospitalizations and limited use of the emergency department [59]. Furthermore, patient activation has proven to be incremental [60, 61]. This makes it even more relevant since it can not only be used for categorizing patients and consumers and tailoring support and education, but also for actual improvement of consumer engagement with respect to their health and health care, both on an individual and on a population level [62–64]. Recognizing that factors at different levels of complexities might affect patient activations, future research should be devoted to enrich the validity of the PAM 13. This could be valuable in order to conduct a more comprehensive assessment of the individual, relational and organizational aspects occurring to foster patient engagement in the care process, as well as to assess clinicians’ communicative and relational practice aimed at sustaining patient activation [15, 65, 66].

## Appendix

**Table 4:** Original PAM13 Questions

PAM13 Item no.	Item
1	When all is said and done. I am the person who is responsible for managing my health condition
2	Taking an active role in my own health care is the most important factor in determining my health and ability to function
3	I am confident that I can take actions that will help prevent or minimize some symptoms or problems associated with my health condition
4	I know what each of my prescribed medications do
5	I am confident that I can tell when I need to go get medical care and when I can handle a health problem myself
6	I am confident I can tell my health care provider concerns I have even when he or she does not ask
7	I am confident that I can follow through on medical treatments I need to do at home
8	I understand the nature and causes of my health condition(s)
9	I know the different medical treatment options available for my health condition
10	I have been able to maintain the lifestyle changes for my health that I have made
11	I know how to prevent further problems with my health condition
12	I am confident I can figure out solutions when new situations or problems arise with my health condition
13	I am confident that I can maintain lifestyle changes like diet and exercise even during times of stress

**Table 5:** PAM13-I Italian translation

PAM13-I item no.	Item	fortemente in disaccordo (1)	in disaccordo (2)	in accordo (3)	fortemente in accordo (4)	n/a
PAM13-I 1	Alla fine sono in prima persona responsabile della gestione del mio stato di salute	○	○	○	○	○
PAM13-I 2	Avere un ruolo attivo nella gestione della mia salute è il fattore più importante nel determinare il mio benessere e la mia qualità di vita	○	○	○	○	○
PAM13-I 3	Sono certo di poter agire in modo da favorire la prevenzione o la riduzione di certi sintomi o problemi legati al mio stato di salute	○	○	○	○	○
PAM13-I 4	Conosco gli effetti di ciascuno delle terapie che mi sono state prescritte	○	○	○	○	○
PAM13-I 5	Sono certo di poter riconoscere quando ho bisogno di cure mediche specialistiche e quando invece posso gestire un problema di salute per conto mio	○	○	○	○	○
PAM13-I 6	So di poter comunicare al mio curante eventuali preoccupazioni inerenti alla mia salute anche se non è lui a chiedermelo	○	○	○	○	○
PAM13-I 7	Sono certo di poter seguire le mie terapie anche da casa	○	○	○	○	○
PAM13-I 8	Conosco le caratteristiche della mia malattia e ciò che l’ha determinata	○	○	○	○	○
PAM13-I 9	Sono a conoscenza delle differenti opzioni terapeutiche disponibili per il trattamento della mia malattia rispetto al mio stato di salute	○	○	○	○	○
PAM13-I 10	Sono stato in grado di mantenere i cambiamenti di stile di vita che ho assunto volti a migliorare la mia salute	○	○	○	○	○
PAM13-I 11	So cosa posso fare per prevenire ulteriori complicanze relativi al mio problema di salute	○	○	○	○	○
PAM13-I 12	So di poter trovare soluzioni nel caso si presentassero situazioni o problemi nuovi connessi al mio stato di salute.	○	○	○	○	○
PAM13-I 13	So di poter mantenere cambiamenti dello stile di vita - quali la dieta o l’esercizio fisico - anche in periodi di stress.	○	○	○	○	○

Di seguito troverai 13 affermazioni che le persone di solito fanno quando parlano della loro salute. Ti chiediamo di indicare quanto ti trovi in accordo o in disaccordo con ciascuna delle affermazioni sulla base della tua esperienza mettendo una X sul numero corrispondente. Qualora trovassi che un’affermazione non ti riguarda, metti invece una X nella colonna n/a

## Abbreviations

DIF: Differential item functioning
MNSQ: Item fit mean square
PCM: Partial credit model
PAM 13: Patient activation measure short form
PSI: Person separation index
PCA: Principal component analysis
RM: Rasch model
